# Editorial: Advances in the treatment of hormonal receptor positive (HR+) breast cancer

**DOI:** 10.3389/fonc.2024.1449566

**Published:** 2024-09-25

**Authors:** Wala Ben Kridis, Zheng Wang, Deniz Can Guven, Arunasalam Dharmarajan

**Affiliations:** ^1^ Habib Bourguiba University Hospital, University of Sfax, Sfax, Tunisia; ^2^ Ruijin Hospital, School of Medicine, Shanghai Jiao Tong University, Shanghai, China; ^3^ Cancer Institute, Hacettepe University, Ankara, Türkiye; ^4^ Medical Centre, Sri Ramachandra Institute of Higher Education and Research, Chennai, India

**Keywords:** breast cancer, CDK 4/6 inhibitors, PIK3CA, genetics, aromatase inhibiters, toxicity

Breast cancer is the most common cancer in women. Breast cancer subtypes are classified according to histologic features, including morphology and receptor status. Information on the expression of estrogen receptor (ER), progesterone receptor, and human epidermal growth factor receptor 2 (HER2), as well as the proliferation index Ki67 (in early-stage disease), are relevant for clinical decisions. Molecular tests are now available to further classify the disease into subgroups, stratify risk or estimate the benefit of interventions. Some examples of such tests include the Recurrence Score (OncotypeDX), PAM50 (Prosigna), Mammaprint, Blueprint, and Breast Cancer Index (BCI), among others.

In recent years, the treatment of HR+HER2- breast cancer has been revolutionized by the introduction of the CDK4/6 inhibitors (CDK4/6i) palbociclib, ribociclib, and abemaciclib, the PI3 kinase inhibitor alpelisib, the antibody drug conjugates trastuzumab-deruxtecan and sacituzumab govitecan, the PARP inhibitor olaparib (when a germline BRCA 1 or 2 mutation is present) as well as the oral selective ER degrader elacestrant for patients with ESR1 mutations.

This Research Topic aims to widen the understanding of the advances in treatment for localized and metastatic HR+HER2- breast cancer to help improve the outcomes for patients. 11 articles were accepted. Starting with aromatase inhibitor and its toxicities. Aromatase inhibitors (AIs) are a cornerstone adjuvant treatment of many hormone receptor-positive breast cancers, and nearly half of women taking aromatase inhibitors suffer from AI-induced arthralgia (AIA), also known as AI associated musculoskeletal syndrome (AIMSS), for which there are limited evidence-based treatments. Pharmacologic management and complementary methods including supplements, exercise, physical therapy, yoga, acupuncture, and massage have all shown mixed results. Comprehensive diet and lifestyle strategies are understudied in AIA/AIMSS despite their disease-modifying effects across many chronic conditions. Wilson et al. reported a case of a woman with stage 2 estrogen and progesterone receptor-positive invasive ductal carcinoma on adjuvant anastrozole whose AI-induced arthralgia was durably controlled through a Mediterranean plant-forward diet and daily physical activity guided by continuous glucose monitoring. They posit that diet and a lifestyle inclusive of daily physical activity constitute a low-cost, low-risk, and potentially high-reward strategy for controlling common AI-induced musculoskeletal symptoms and that more investigation in this arena, including well-designed randomized trials, is warranted. Chu et al. aimed to establish a high-risk prediction model for aromatase inhibitor associated bone loss (AIBL) in patients with hormone receptor-positive.

The identified risk factors were used to construct a prediction model using the eXtreme gradient boosting (XGBoost) machine learning method. Logistic regression and least absolute shrinkage and selection operator (LASSO) regression methods were used for comparison. A total of 113 subjects were included in the study. Time from diagnosis of breast cancer, duration of aromatase inhibitor therapy, hip fracture index, major osteoporotic fracture index, prolactin (PRL), and osteocalcin (OC) were found to be independent risk factors for AIBL (p < 0.05). The XGBoost model had a higher AUC compared to the logistic model and LASSO model (0.761 vs. 0.716, 0.691). Consequently, authors concluded that the XGBoost model outperformed the logistic and LASSO models in predicting the occurrence of AIBL in patients with hormone receptor-positive breast cancer receiving aromatase inhibitors.

Previous studies have shown that osteoporosis is a side effects of the breast cancer hormone therapy, although the exact mechanisms remain mostly unclear. Current clinical treatments, such as bisphosphonates, cause side effects and may impact the therapeutic response to endocrine drugs.

According to Xu et al. traditional Chinese medicine has great potential in the prevention and treatment of osteoporosis caused by endocrine therapy in breast cancer. For instance, isosinensetin, a flavonoid present in citrus fruits with antioxidant properties, has been shown to reduce bone loss in OVX mice and alleviate estrogen deficiency-induced osteoporosis in mice. Obacunone, a small molecule with a wide range of biological activities, can inhibit the formation and absorption function of OCs *in vitro* by targeting inhibitory factor of macrophage migration inhibitory factor (MIF). Thus, it is expected to be an effective drug for relieving osteoporosis caused by estrogen deficiency. It is necessary to explore their relationship with osteoporosis to provide new treatment strategies. The relationship between bone lymphatics and osteoporosis remains to be further explored. Additionally, the pathogenesis of breast cancer is not yet fully understood, and there is a need for more effective therapeutic drugs to reduce the occurrence of adverse reactions related to endocrine therapy.

In recent years, significant strides have been made in the management of HR+/HER2- breast cancer through the introduction of CDK4/6 inhibitors, such as palbociclib, ribociclib, and abemaciclib, thereby improving outcomes for adjuvant, advanced and/or metastatic settings. CDK4/6 inhibitors can block retinoblastoma protein hyperphosphorylation, inducing G1 arrest and curtailing proliferation.

Rodrigues Alves et al. reported the first case of a patient presenting with bilateral orbital metastases from bilateral lobular breast cancer, showing an impressive and sustained response to a first-line treatment regimen combining abemaciclib and letrozole. Orbital metastases represent 1-13% of all orbital neoplasm and affecting around 2-5% of patients with cancer. They are typically unilateral. Bilateral orbital metastases are reported in 4%. They are often identified after the primary tumor. However, in the case of Rodrigues Alves et al. they revealed the diagnosis of bilateral breast cancer without any other metastatic site. Furthermore, this is the first case of bilateral orbital metastases from bilateral breast cancer treated by abemaciclib and letrozole with complete response in both breasts and significant improvement on orbital imaging with good visual acuity. In case of progression after hormone therapy with CDK4/6 inhibitors, Elascestrant is indicated in case of ESR1 mutation. Zeng et al. evaluated the cost-effectiveness of elacestrant (ELA) and standard-of-care (SOC) as second-/third-line treatment for pretreated estrogen receptor (ER)– positive/human epidermal growth factor receptor 2 (HER2)– negative advanced or metastatic breast cancer (A/MBC) in the US. They concluded that ELA was not cost-effective for the second-/third-line treatment of patients with ER+/HER2–A/MBC compared with SOC in the US. In fact, ELA led to an incremental cost-effectiveness ratio (ICER) of $8,672,360/quality-adjusted life year (QALY) gained compared with SOC in the overall population and $2,900,560/QALY gained compared with fulvestrant (FUL) in the ESR1(estrogen receptor 1) mutation subgroup.

Consistent with previous studies, Chao et al. identified a high prevalence of PIK3CA mutations in 38% of the Taiwanese patients with breast cancer. The lower prevalence in premenopausal patients and patients with triple-negative breast cancer warrants further studies. Most of the mutations were in exon 9 and exon 20, with H1047R, E545K, and E542K being the hotspots. A longer time to treatment failure in wild-type PIK3CA cohorts treated with CDK4/6 inhibitors was reported, which demonstrated the better efficacy of CDK4/6 inhibitors in wild-type PIK3CA cohorts than that in the PIK3CA-mutant cohort. Everolimus, an mTOR inhibitor, reported a longer time to treatment failure in the PIK3CA-mutant cohort and demonstrated better efficacy.

Concerning the place of genetics in the diagnosis of breast cancer, AGR2 is a secreted protein widely existing in breast. Its endoplasmic reticulum retention sequence, protein disulfide isomerase active site and multiple protein binding sequences endow AGR2 with diverse functions inside and outside breast cancer cells. Zhang et al. concluded in their review that diagnostic tools such as microfluidic detection devices or biosensors can be developed to detect AGR2 specifically and sensitively. Combining AGR2 with other tumor markers can improve the sensitivity of breast cancer diagnosis, which is one of the hot spots that clinicians need to pay attention to in the future. So far, therapeutic strategies targeting AGR2 have shown promising results. For example, by constructing the bispecific antibodies of AGR2 antibody and immune checkpoint proteins, it can play its role in tumor tissue with maximum target concentration, which is a clinical transformation direction to improve the efficacy and reduce side effects.

Cancer-associated fibroblasts (CAFs) play a pivotal role in cancer progression and are known to mediate endocrine and chemotherapy resistance through paracrine signaling. Additionally, they directly influence the expression and growth dependence of ER in Luminal breast cancer (LBC). Xu et al. aimed to investigate stromal CAF-related factors and develop a CAF-related classifier to predict the prognosis and therapeutic outcomes in LBC. They constructed a 5-gene prognostic model consisting of RIN2, THBS1, IL1R1, RAB31, and COL11A1 for CAF. Gene set enrichment analysis (GSEA) identified significant enrichment of ECM receptor interaction, regulation of actin cytoskeleton, epithelial-mesenchymal transition (EMT), and TGF-b signaling pathway gene sets in the high-CAF-risk group patients. Then, they concluded that the five-gene prognostic CAF signature presented in this study was not only reliable for predicting prognosis in LBC patients, but it was also effective in estimating clinical immunotherapy response. These findings have significant clinical implications, as the signature may guide tailored anti-CAF therapy in combination with immunotherapy for LBC patients.

Endeavors in the molecular characterization of breast cancer opened the doors to endocrine therapies in ER+/HER2- breast cancer, increasing response rates substantially. Despite that, taxane-based neoadjuvant chemotherapy is still a cornerstone for achieving breast-conserving surgery and complete tumor resection in locally advanced cancers with high recurrence risk.

Nonetheless, the rate of chemoresistance is high, and deselecting patients who will not benefit from chemotherapy is a significant task to prevent futile toxicities. Several multigene assays are being used to guide decisions on chemotherapy. However, their development as prognostic assays but not predictive assays limits predictive strength, leading to discordant results. Moreover, high costs impediment their use in developing countries. For global health equity, robust predictors that can be cost-effectively incorporated into routine clinical management are essential. Protein patched homolog 1 (PTCH1) is a member of the patched gene family and is the receptor for sonic hedgehog, a secreted molecule implicated in the formation of embryonic structures and in tumorigenesis. This gene functions as a tumor suppressor. β-Catenin (CTNNB1) is a dual function protein, involved in regulation and coordination of cell–cell adhesion and gene transcription. Ozcan suggests that PTCH1 and CTNNB1 can be used as robust and cost-effective predictors in developing countries to guide decisions on chemotherapy in ER +/HER2- breast cancer patients with a high risk of recurrence. The dual function of PTCH1 as a multidrug efflux pump and a hedgehog receptor, and the active involvement of CTNNB1 in breast cancer strongly indicate that PTCH1 and CTNNB1 can be potential drug targets to overcome chemoresistance in ER +/HER2- breast cancer patients.

The lethal-7 (Let-7i) family is an important microRNA (miRNA) group that usually exerts functions as a tumor suppressor. According to Zhou et al. Let-7i regulates tumors primarily by binding to the 3′ untranslated region (3′ UTR) of mRNA, which indirectly regulates post-transcriptional gene expression. Let-7i also has an epigenetic function via modulating DNA methylation to directly regulate gene expression. Let-7i performs a dual role by inducing both the promotion and inhibition of various malignancies, depending on its target. The mechanism of Let-7i action involves cancer cell proliferation, migration, invasion, apoptosis, epithelial-mesenchymal transition, EV transmission, angiogenesis, autophagy, and drug resistance sensitization. Let-7i is closely related to cancer, and hence, is a potential biomarker for the diagnosis and prognosis of various cancers. Therapeutically, it can be used to promote an anti-cancer immune response by modifying exosomes, thus exerting a tumor-suppressive effect.

We can conclude in this Research Topic that diet and a lifestyle inclusive of daily physical activity constitute a low-cost, low-risk, and potentially high-reward strategy for controlling common AI-induced symptoms. Furthermore, traditional Chinese medicine has great potential in the prevention and treatment of osteoporosis caused by endocrine therapy in breast cancer. XGBoost model outperformed the logistic and LASSO models in predicting the occurrence of AIBL in patients with hormone receptor-positive breast cancer receiving aromatase inhibitors. Therapically, elacestrant was not cost-effective for the second-/third-line treatment of patients with ER+/HER2–A/MBC compared with SOC in the US. Concerning patients with PIK3CA-mutation, Everolimus reported a longer time to treatment failure and demonstrated better efficacy. Regarding the place of genetics in the diagnosis of breast cancer, combining AGR2 with other tumor markers can improve the sensitivity of breast cancer diagnosis. Also, RIN2, THBS1, IL1R1, RAB31, and COL11A1 for CAF were not only reliable for predicting prognosis in LBC patients, but they were also effective in estimating clinical immunotherapy response. Finally, Let-7i can be used to promote an anti-cancer immune response by modifying exosomes, thus exerting a tumor-suppressive effect.

